# Surface Passivation Treatment to Improve Performance and Stability of Solution‐Processed Metal Oxide Transistors for Hybrid Complementary Circuits on Polymer Substrates

**DOI:** 10.1002/advs.202101502

**Published:** 2021-10-20

**Authors:** Moon Hyo Kang, John Armitage, Zahra Andaji‐Garmaroudi, Henning Sirringhaus

**Affiliations:** ^1^ Optoelectronics Group Cavendish Laboratory University of Cambridge J J Thomson Avenue Cambridge CB3 0HE UK; ^2^ 2D Materials and Devices Group Department of Materials Science & Metallurgy University of Cambridge 27 Charles Babbage Road Cambridge CB3 0FS UK; ^3^ 412 Riverdale Avenue Ottawa Ontario K1S1S2 Canada

**Keywords:** air stability, analog differential amplifier, high mobility, oxygen vacancy, solution‐processed metal oxide transistors

## Abstract

Hybrid integration of n‐type oxide with p‐type polymer transistors is an attractive approach for realizing high performance complementary circuits on flexible substrates. However, the stability of solution‐processed oxide transistors is limiting the lifetime and reliability of such circuits. Oxygen vacancies are the main defect degrading metal oxide transistor performance when ambient oxygen adsorbs onto metal oxide films. Here, an effective surface passivation treatment based on negative oxygen ion exposure combined with UV light is demonstrated, that is able to significantly reduce surface oxygen vacancy concentration and improve the field effect mobility to values up to 41 cm^2^ V^−1^ s^−1^ with high on–off current ratio of 10^8^. The treatment also reduces the threshold voltage shift after 2 days in air from 5 to 0.07 V. The improved stability of the oxide transistors also improves the lifetime of hybrid complementary circuits and stable operation of complementary, analog amplifiers is confirmed for 60 days in air. The suggested approach is facile and can be widely applicable for flexible electronics using low‐temperature solution‐processed metal oxide semiconductors.

## Graphical abstract

Combined treatment by ultraviolet radiation and negative oxygen ions can effectively passivate oxygen vacancies on the surface of metal oxide films, resulting in significantly improved field‐effect mobility and stability. It enables complementary analog amplifiers based on n‐type metal oxide and p‐type polymer transistors with improved performance and lifetime. The simple passivation approach is widely applicable for solution‐processed oxide electronics.

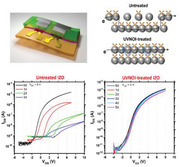

## 1 Introduction

Organic semiconductors and amorphous metal oxide semiconductors are intensely researched for applications in flexible analog electronics. P‐type conjugated polymers have advanced greatly in performance and reliability as a channel layer of field effect transistors (FETs) in flexible electronics, but n‐type polymer materials still suffer from relatively poorer ambient stability,^[^ ^1^ ^]^ making realization of complementary circuits challenging.^[^ ^2^ ^]^ For metal oxide semiconductors, the situation is reversed, n‐type channel devices are more readily realized than p‐type device.^[^ ^3^ ^]^ Unipolar circuits based on either p‐type or n‐type devices only suffer from poor voltage gain and lower noise margin^[^ ^4^, ^5^, ^6^, ^7^ ^]^ as well as higher power consumption. Highly performing complementary circuits can be realized by employing a combination of solution‐processed p‐type polymer semiconductors and n‐type metal oxide semiconductors on the same substrate, making use of the high hole mobility of state‐of‐the‐art polymer semiconductors (1–5 cm^2^ V^−1^ s^−1^) and the high electron mobility of metal oxide materials (1–50 cm^2^ V^−1^ s^−1^).^[^ ^8^ ^]^ Adopting the best of both worlds, we formerly demonstrated a complementary differential amplifier combining solution‐processed p‐type poly(indacenodithiophene–benzothiadiazole) (IDT–BT) and solution‐processed n‐type indium zinc oxide (IZO), which can be operated at low voltage (<10 V).^[^ ^9^ ^]^

However, the combination of such different types of semiconducting materials introduces challenging processing constraints when integrating the materials onto a common substrate, particularly when using a flexible substrate that is subject to process temperature constraints.^[^ ^10^ ^]^ Process conditions for the second material (e.g., process temperature, choice of solvents, or conditions for semiconductor patterning) can have influence on the previously deposited material. In particular, environmental/air stability is a critical factor that can be compromised when trying to find process conditions that work for both materials. The air stability of the p‐type polymer, IDT–BT, can simply be improved by incorporating a small organic molecule additive, such as tetrafluoro‐tetracyanoquinodimethane (F4‐TCNQ) or tetracyanoquinodimethane into the polymer film.^[^ ^9^, ^11^ ^]^ However, the air instability of low‐temperature, solution‐processed metal oxide thin films is more challenging to address. It manifests itself as a large threshold voltage (*V*_th_) shift when devices are exposed to ambient oxygen or water molecules.^[^ ^12^, ^13^, ^14^ ^]^

In solution‐processed oxide semiconductors, oxygen vacancies (V_O_) associated with the difficulty of controlling the film stoichiometry when converting the chemical precursors at moderately elevated temperature have been recognized as the main defect determining the stability under ambient conditions. Deep carrier trap levels can be created ≈0.5 eV below the conduction band minimum (CBM) by the ionization of oxygen vacancies, resulting in generation of free electrons and the vacancy defects becoming positively charged.^[^ ^15^, ^16^, ^17^ ^]^ When such films are exposed to oxygen (O_2_) from ambient air, the adsorbed oxygen molecule may become superoxide, $O_{2}^{-}$ at room temperature by accepting a free electron near film surface.^[^ ^18^, ^19^, ^20^ ^]^ The $O_{2}^{-}$ may migrate to the bulk through loosely bonded sites or vacancies and dissociate to form O^−^ by taking more free electrons.^[^ ^20^ ^]^ As a result, both O^−^ and $O_{2}^{-}$ species may coexist near the surface. When O^−^ eventually reacts with the metal in oxygen vacancy sites and becomes lattice oxygen, O^2−^ , this process will have led to the capture/trapping of four free electrons per adsorbed O_2_ and will result in an increase in FET threshold voltage

(1) $$\begin{array}{c} 2M^{2+}+2V_{O}+4e^{-}+O_{2}\left(\mathrm{Ambient}\right)\to 2M^{2+}+2O^{2-}\left(\mathrm{ionic}\;\;\mathrm{bond}\right) \end{array}$$

In sputter‐deposited oxides, an effective way for stability improvement is to alter the stoichiometry of oxygen ions by controlling oxygen vapor pressure in the deposition atmosphere.^[^ ^21^, ^22^ ^]^ Oxygen deficiency in metal oxide films is able to be reduced by an additional oxygen gas flow during the film formation and the vertically controlled oxygen ratio improves FET mobility and stability.^[^ ^23^ ^]^ Recent thermal crystallization approach also improves oxygen stoichiometry, resulting in enhanced carrier mobility and electrical stability.^[^ ^24^ ^]^ However, these approaches are not directly applicable to solution‐processed metal oxide films due to the difficulty in adjusting oxygen concentration during sol–gel processing in ambient air. As a result, solution‐processed metal oxide films tend to contain more oxygen vacancies compared to films deposited by sputtering.^[^ ^25^ ^]^ To reduce vacancy concentrations, approaches such as doping, high pressure annealing, and multilayer stacking have been pursued and are listed in Table S1 (Supporting Information). Most of the listed studies adopted zinc oxide doped with indium (In) or gallium (Ga). Indium has smaller oxygen vacancy formation energy and larger s‐orbital radius compared to zinc or gallium,^[^ ^25^ ^]^ indium‐doped zinc oxide typically has higher carrier mobility and higher carrier concentration with additional free electrons delivered from oxygen vacancies. Gallium forms strong bonds with oxygen, this reduces carrier concentrations due to higher vacancy formation energy. Generally, approaches to reduce *V*_th_ shifts in metal oxide films by suppressing metastable oxygen vacancy states also tend to reduce carrier concentration in the films. This can diminish performance of FETs if there is an insufficient free carrier concentration to compensate deep electron trap states. If these defects then are filled with field‐induced electrons, this leads to large threshold voltages. Here, we investigate a surface passivation approach that simultaneously improves the air stability of solution‐processed n‐type IZO FETs, but also enhances carrier mobility and make these oxide devices suitable for integration with p‐type IDT–BT FETs into air‐stable analog differential amplifiers. The approach can be applied to both sputtered and solution‐processed metal oxide film and the room‐temperature treatment is compatible with organic fabrication processes on flexible substrates.

## 2 Results and Discussion

Metal oxide films need to be annealed at temperatures higher than 250 °C for the complete decomposition of precursors and dehydration during the annealing.^[^ ^26^ ^]^ Such high temperature is a harsh condition for the p‐type polymer channel layer and thus the metal oxide film should be formed before the deposition of the other layers of the organic–inorganic hybrid complementary structure. To be compatible with the preferred top‐gate architecture of the polymer FET, we use a top‐gate device architecture for the oxide FET as well. In this structure, the conducting FET channel is formed on the top surface of the metal oxide film which contains more oxygen vacancies and is more likely to interact with oxygen molecules adsorbed from the air.^[^ ^27^ ^]^ Also, we used a common gate dielectric formed from a solution‐processed polymer dielectric which does not provide an effective encapsulation against oxygen ingress. When positive bias is applied to the gate to turn on the channel, free electrons in the metal oxide film are accumulated on the top surface, i.e., the interface between the metal oxide and the gate dielectric layer. The accumulation of electrons might assist the conversion of physisorbed O_2_ into $O_{2}^{-},O^{-},$ or O^2−^ , resulting in electron trapping and threshold voltage shifts. Our selected architecture for integration is therefore particularly sensitive to air‐induced instabilities and requires particularly effective strategies for passivating defect states in the oxide semiconductor.

**Figure 1:**
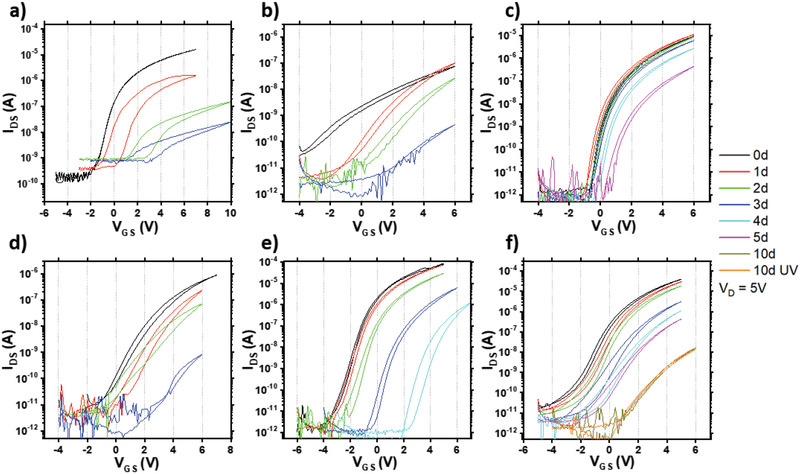
IZO FET transfer characteristics measured as a function of time while being stored in air for different treatments of the IZO film: a) untreated IZO, b) Li‐doped IZO, c) F‐doped IZO, d) H_2_O_2_‐treated IZO, e) UV + ozone‐treated IZO, and f) UV + O_2_‐plasma‐treated IZO.

All IZO FETs in our study were fabricated on polyimide substrates using a sol–gel process with metal‐salt precursors, as described in the Experimental Section. To facilitate low‐voltage driving circuits, a solution‐processable high‐*k* relaxor ferroelectric (*k* ≈ 40), poly(vinylidene fluoride–trifluoroethylene–chlorofluoroethylene) (P(VDF–TrFE–CFE)) was used as a gate dielectric in combination with a low‐*k* interfacial dielectric (CYTOP) to ensure a high carrier mobility, particularly of the polymer FET. The electrical performances of the dielectrics, CYTOP and P(VDF–TrFE–CFE) have been reported elsewhere.^[^ ^9^, ^28^ ^]^ Large dipole moment of the high‐*k* ternary copolymer enables strong gate‐to‐channel capacitive coupling, enabling low voltage FET operation. Interfacial dipolar disorder due to the high‐*k* polymer ferroelectric can be avoided by inserting low‐*k* dielectric polymer, CYTOP. Aluminum was thermally deposited as a gate metal through a shadow mask on the top of the dielectric layer. Untreated IZO FETs (**Figure** **1a**) exhibited a strong air‐induced instability and a large 5 V *V*_th_ shift in their transfer characteristics (*I*_D_–*V*_G_ curve) and an on‐current (*I*_on_) decrease from 16 µA to 42 nA was observed after 2 days storage in air. We also detect an increase in the (clockwise) hysteresis of the transfer characteristics to ≈1.8 V wide after 2 days. The degradation is attributed to the trapping of electrons by adsorbed ambient oxygens as explained above. The trapped electrons cannot be instantly released, inducing the clockwise hysteresis. During subsequent measurements, the degraded current level does not recover, indicating that some of the accumulated electrons are deeply trapped (Figure S1, Supporting information).

To improve the device stability, we attempted implementing various literature treatments in the processing of the IZO film. The corresponding transfer characteristics during air exposure are shown Figure 1. We evaluated Li doping of the films expecting that Li incorporation may assist the formation of a denser film with better arranged metal–oxygen stoichiometric (M—O) bonds and less oxygen vacancies.^[^ ^29^, ^30^ ^]^ However, our Li‐doped IZO FETs had low *I*_on_ (75 nA) and small on–off current ratio (≈10^3^) with hysteresis even on the first day of measurement (Figure 1b). F‐doping of the IZO films to reduce oxygen vacancies was also investigated as F ions have similar ionic size and can substitute for lattice oxygens during the film formation process.^[^ ^31^ ^]^ The substitution creates a free electron due to the difference in the electrovalence of F^−^ and O^2−^. Although F‐doped films exhibit indeed more stable FETs (Δ*V*_th_ = 2.3 V and *I*_on_ decreasing from 11 µA to 300 nA at *V*_G_ = 6 V after being stored in air for 5 days) than our reference untreated IZO FET, there was still too large degradation for the integration into complementary circuits. (Figure 1c). There were previous reports on metal oxide film fabrication using hydrogen peroxide (H_2_O_2_) which is thermodynamically unstable and decomposes to water and oxygen.^[^ ^32^, ^33^ ^]^ H_2_O_2_ can assist sol–gel metal oxide film formation by decreasing annealing temperature with its reactivity and scavenging organic residues or hydrolyzed metal. The devices (Figure 1d) showed no improvements in stability and severe degradation in *I*_on_ (<1 nA at 6 V *V*_G_ after 5 days). As displayed in Figure 1e,f, ozone and weak O_2_ plasma surface treatments were also tried on IZO films. However, the *V*_th_ shift of the FETs were large (Δ*V*_th_ozone‐treated_ = ≈6 V and Δ*V*_th_plasma‐treated_ = ≈2.3 V) after 4 days. In our evaluation, none of the evaluated literature treatments were effective in suppressing the device instability.

In the neutral charge state of the oxygen vacancy (V_O_^0^), the metal atoms energetically relax inward toward the middle of vacancy and this induces a deep‐level defect having its Fermi level (*E*_F_) set near the valence band maximum (VBM) which cannot contribute to n‐type conductivity. However, in ionized vacancies (V_O_^+^ and V_O_^2+^), the metal atoms slightly relax outward and can release electrons.^[^ ^34^ ^]^ V_O_^0^ is thermodynamically stable at room temperature, but it is evident that ionization can take place with some external energy, overcoming the ionization potential of the vacancies and this has been utilized for improving n‐type conductivity.^[^ ^35^, ^36^, ^37^ ^]^ Additionally, the released electrons from V_O_^2+^ do not easily recombine or only very slowly.^[^ ^38^ ^]^ For ozone and plasma‐treated samples (Figure 1e,f), we illuminated the IZO film with a UV light source (365 nm) before treatments to provide some external energy for oxygen vacancy ionization. Neutral vacancies are predicted to transform to positively charged vacancies with slightly relaxed M—O bonds by exposure to UV light. The possibility of breaking some bonds in the film with the UV illumination can also not be excluded. The treated samples showed initially negative *V*_th_ (UV + ozone‐treated: −1.8 V, UV + plasma‐treated: −0.7 V) which reflects the increase in electron concentration generated by V_O_^2+^ formation. However, the channel conductivity kept decreasing with ambient oxygen diffusion to the film for several days. We also attempted to reset degraded devices by performing UV illumination on the device after storage in air for 10 days. However, this did not lead to a recovery (Figure 1f).

**Figure 2:**
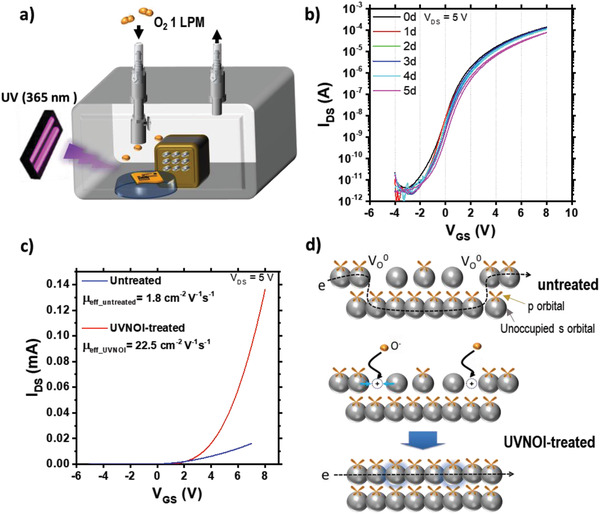
a) Schematic diagram of UVNOI treatment on IZO film, b) transfer characteristics of the IZO FET fabricated with UVNOI treatment, c) transfer characteristics comparison between the untreated and UVNOI‐treated IZO FETs, and d) a schematic diagram of the potential surface structure with oxygen vacancies in pristine IZO channel, that are being passivated by the UVNOI treatment.

To achieve a more effective stability improvement, we propose here a novel combined ultraviolet light–negative oxygen ion (UVNOI) surface passivation treatment. **Figure** **2a** illustrates the UVNOI treatment in an O_2_‐flowing chamber with a negative ion generator (Ion trading – Universal Plan Co. Ltd.) with the detailed recipe specified in the Experimental Section (UVNOI Treatment). The main purpose of the negative oxygen ion (NOI) is to fill up the oxygen vacancy sites on the surface and prevent electron trapping. We hypothesized that if the top surface has an oxygen‐rich stoichiometric composition without oxygen vacancies, the bulk of the film may be less exposed to ambient oxygen due to blocking of diffusion paths. Moreover, the oxygen species generated by the NOI process are already negatively charged and therefore do not trap as many electrons from the film to become lattice oxygen. Therefore, NOI can accomplish a passivation effect on the surface without decrease in electron concentration. We also hypothesize that the simultaneous UV illumination may generate positively charged oxygen vacancies on surface and the associated outward structural relaxation may provide more space for NOI to easily fill up the vacancies and/or the Coulombic attraction force between the ionized vacancy and the NOI may facilitate formation of M—O bonds on surface according to:

(2) $$\begin{array}{c} M^{2+}+V_{O}^{2+}+2e^{-}+O^{-}\left(\mathrm{NOI}\right)\to M^{2+}+O^{2-}\left(\mathrm{ionic}\;\;\mathrm{bond}\right)+1e^{-} \end{array}$$

Therefore, even if the reaction of adsorbed NOI proceeds all the way to the full healing of oxygen vacancies as described by Equation (2), this can occur with less reduction in free carrier concentration compared to the ambient oxygen reaction described by Equation (1). In addition, the positively charged oxygen vacancies release free electrons to the film, resulting in an increase of net carrier concentration. The treatment also offers electrical neutralization of the surface by passivating the oxygen vacancies so that ambient oxygens have less chances to be attracted by Madelung potential of the surface. Hence, the NOI‐treated surface can effectively prevent ambient oxygen adsorption and diffusion to the inside of the film.

With this in mind, we subjected IZO FETs to the combined UVNOI treatment and measured their *I*–*V* characteristics for 5 days in air. All information on device geometry, layer thickness, and fabrication procedure are described in the Experimental Section. As shown in the transfer curve of UVNOI‐treated IZO FET (Figure 2b), the on–off current ratio (≈10^8^) was notably larger than that (≈10^5^) of untreated IZO FET (Figure 1a). The field effect (FE) mobility of UVNOI‐treated and untreated IZO (Figure 2c) were extracted from the transistor characteristics using a reliable, conservative method, as detailed in Figure S2 (Supporting Information). The extracted mobility of UVNOI increases with increasing *V*_GS_ up to 15 V (Figure S2b, Supporting Information). In such cases, the reliability factor (*r*) should be considered to extract the conservatively estimated effective carrier mobility (*μ*_eff_  =  *r*  ×  *μ*_sat_).^[^ ^39^ ^]^ The effective mobility was extraordinarily improved by the treatment from 1.8 cm^2^ V^−1^ s^−1^ (untreated) to 22.5 cm^2^ V^−1^ s^−1^ (UVNOI‐treated) at 7 V. The corresponding maximum value of FE mobility extracted from UVNOI device was 41.2 cm^2^ V^−1^ s^−1^ at 15 V (Figure S2, Supporting Information) which is 21 times higher than the maximum mobility at 1.8 V from the untreated device (1.9 cm^2^ V^−1^ s^−1^). Even with the application of the reliability factor, the mobility of UVNOI devices is 12 times higher than that of the untreated ones. We hypothesize that the improvement in mobility can be attributed to two effects: UV illumination generates additional free electrons and thus the treated IZO films have a higher free electron concentration available to fill shallow electron trap levels than the undoped films. Moreover, the large mobility enhancement could also reflect a rearrangement of M—O bonds on the IZO surface induced by UVNOI treatment. There are no grain boundaries in amorphous metal oxide semiconductors, but surface areas with small pores or higher oxygen vacancy concentration may act as scattering centers for electron transport.^[^ ^40^ ^]^ The treatment is likely to induce a denser network of M—O bonds on the surface, facilitating faster electron transport with reduced surface scattering (Figure 2d).

As explained above, the air instability of solution‐processed IZO films is primarily attributed to electron trapping by ambient oxygen molecules adsorbed on the surface and ambient oxygen is more likely to be adsorbed on the surface when the surface has a large fraction of low coordinated cations because unstable cations tend to be stabilized by bonding with the adsorbed oxygen.^[^ ^41^ ^]^ UVNOI can prepare a more neutral surface with fewer oxygen vacancies and reduce the attractive Madelung potential between ambient oxygen molecules and surface metal cations so that the probability of oxygen adsorption on the surface is reduced and in this way the surface is passivated. The on‐current of UVNOI‐treated IZO FET exhibits a comparably much reduced decrease from 163 to 76 µA at 7 V *V*_G_ after 5 days (Figure 2b), while the untreated IZO FET exhibited a large reduction in the current level from 9 µA to 3 nA at the same *V*_G_ (Figure 1a). With the treatment, the *V*_th_ shift was negligibly small (Δ*V*_th_ = 0.07 V) from 1.03 to 1.1 V after 2 days and 0.5 V shift was observed even after 5 days, indicating substantial stability improvement from the untreated (Δ*V*_th_ = 5 and 8 V after 2 and 5 days, respectively).

**Figure 3:**
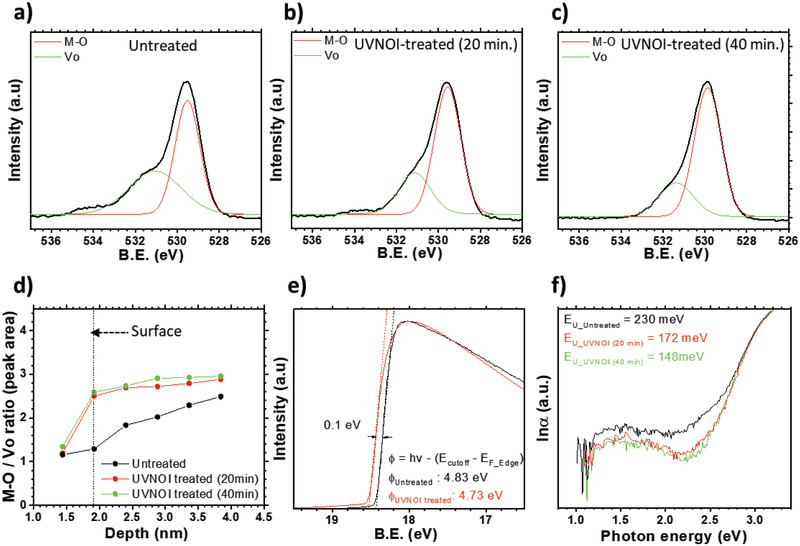
XPS O1s peaks of the film surface from a) untreated IZO, b) 20 min UVNOI‐treated IZO, c) 40 min UVNOI‐treated IZO, d) depth profile of O1s peak; the peak area ratio of fully coordinated oxygen with metal (M—O) and oxygen vacancy (V_O_) as a function of etched depth by Ar ion. e) Secondary UPS cutoff and f) PDS analysis of untreated and UVNOI‐treated IZO films. The absorption spectra were used to calculate the Urbach energy which is a measure of the energetic disorder near the band edges in an amorphous material.

In order to obtain analytical information on the stability‐enhancing effect of the surface treatment, X‐ray photoemission spectra (XPS) analysis was performed with untreated and UVNOI‐treated IZO films. Analysis of the chemical shift of the O1s core level can reveal oxygen bonding configuration with neighboring atoms in the film and the depth profile of O1s can reveal how the concentration of oxygen vacancies varies from surface to bulk. XPS of the very top surface layer is not easily acquired because the X‐ray penetration depth is not zero but on the order of 1 nm.^[^ ^42^ ^]^ Hence, a thin layer of our interfacial dielectric CYTOP (4–5 nm) was coated on the IZO film surface and multiple measurements were executed after etching in steps of ≈0.24 nm depth by Ar‐ion sputtering. The O1s peak from CYTOP exhibits a peak from fluorine‐bonded oxygen (O—F) at ≈535 eV.^[^ ^43^ ^]^ When this peak disappears during depth profiling and the M—O bonding peak at 529.5 eV^[^ ^44^ ^]^ becomes stronger, this indicates that the IZO film surface has been exposed. The O1s peak was fitted with a Voigt convolution taking into account three components of oxygen bonding (M—O, V_O_, and O—F), as shown in Figure S4 (Supporting Information). **Figure** **3a**–c presents the O1s analysis of untreated, 20 min UVNOI‐treated, and 40 min UVNOI‐treated IZO films at the same etching depth of 1.92 nm. The depth was supposed to be the IZO film surface as a strong M—O bonding peak was first observed, while the O—F peak was much reduced. In Figure 3a, the pronounced hump shape with a side peak 531.2 eV is from oxygen vacancies (V_O_).^[^ ^45^, ^46^ ^]^ In comparison, the UVNOI‐treated IZO (Figure 3b,c) showed significantly smaller V_O_ peaks and larger fully coordinated M—O peaks. The results demonstrate that the treatment can give rise to less amount of oxygen vacancies on surface.

The ratio between M—O‐bonded oxygens and V_O_ can be calculated from the relative peak area of the main components as a function of depth (Figure 3d). The starting depth of 1.44 nm is near the interface of CYTOP and IZO but has not yet exposed the surface of IZO as it still has fluorine peaks and weaker M—O peak (Figure S4, Supporting Information). Hence, the ratio values at this depth are not very meaningful (1.1–1.3). However, on the film surface (1.92 nm depth), the M—O/V_O_ ratios in the UVNOI‐treated IZO film were 2.50 (20 min treated) and 2.58 (40 min treated), respectively, which are double the value measured on the untreated surface (1.29). This directly manifests the reduction in oxygen vacancy concentration on the surface and increased M—O bond formation when IZO is treated with UVNOI. Despite the low M—O/V_O_ ratio of untreated IZO at the surface, the ratio increases toward the bulk, indicating that more oxygen vacancies exist on the surface of untreated IZO than in the bulk. This is attributed to a lower vacancy formation energy on the surface due to reduced metal coordination.^[^ ^44^, ^45^ ^]^ By contrast, the M—O/V_O_ ratio of UVNOI‐treated IZO film showed a nearly flat depth profile, suggesting that the surface was successfully passivated and composed of stable M—O bonds. This is fully consistent with the observed improvements of device stability and mobility discussed above. We also measured the film work function using ultraviolet photoemission spectra (UPS) with a He I lamp (21.2 eV). As shown in Figure 3e, the work function was reduced by 0.1 eV from 4.83 to 4.73 eV by the treatment. The work function change reflects a Fermi level (*E*_F_) shift toward the CBM, which is consistent with the removal of electron traps by the UVNOI process.

Photothermal deflection spectroscopy (PDS) was performed to probe defect states and their associated sub‐bandgap optical absorptions. PDS is a very sensitive optical absorption technique based on measuring the heat that is dissipated when sub‐bandgap light is being absorbed by the sample.^[^ ^46^, ^47^, ^48^ ^]^ In amorphous materials, structural disorder causes self‐localization of carriers and the spectral dependence of the optical absorption edge can be described as

(3) $$\alpha =\alpha_{0}\exp\left(\left(h\nu -E_{g}\right)/E_{U}\right)$$

where *α*_0_ is a constant and *E*_U_ is the Urbach energy which is a measure of the energetic disorder in the joint density of states between the tails of the VBM and the CBM^[^ ^49^ ^]^ (Figure 3f). In the untreated films, we observed a relatively wide Urbach tail with *E*_U_untreated_ = 230 meV, while the UVNOI‐treated films exhibit a lower Urbach energy of *E*_U_20 min treated_ = 172 meV and *E*_U_40 min treated_ = 148 meV after 20/40 min, respectively. In the UVNOI‐treated films, we also observed a reduction in the broad sub‐bandgap absorption band centered around 1.5 eV, which might be due to excitation of electrons from deep occupied defect levels above the VBM to empty states near the CBM. These observations indicate a reduced density of deep levels and shallow tail states upon UVNOI treatment and are fully consistent with the reduced oxygen vacancy concentration deduced from XPS and the observed improvement in FET performance and stability. Moreover, the results of successful oxygen vacancy reduction with UVNOI surface passivation also suggest the possibility for operational stability improvement in UVNOI‐treated FETs. The prolonged application of a positive gate bias is likely to lead to deep trapping of electrons by oxygen vacancies, as verified in Figure S1 (Supporting Information). The suppression of oxygen vacancy can prevent electrons from being trapped and thus, improved bias stability is expected with the UVNOI treatment. A detailed investigation of FET bias stability such as positive bias stress and negative bias stress will be explored in our forthcoming research.

**Figure 4:**
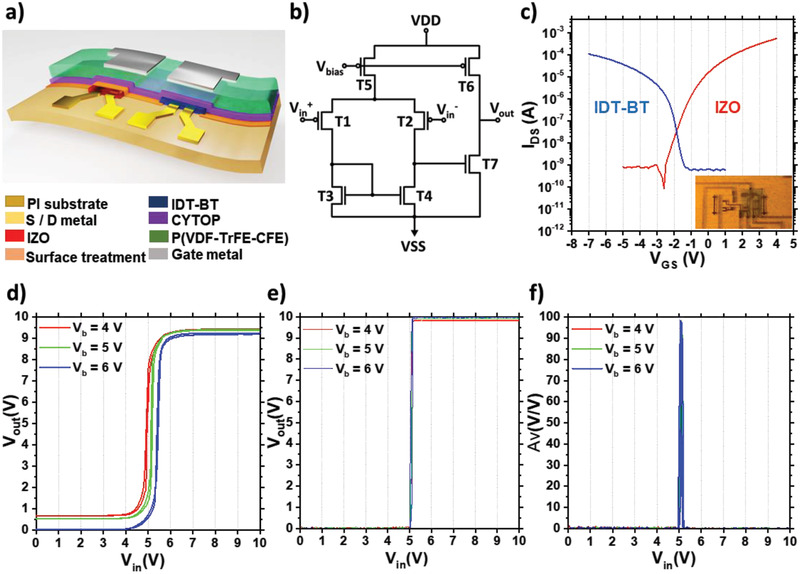
Differential amplifier on flexible substrate using solution‐processed organic and metal oxide hybrid complementary FETs. a) Cross‐sectional view of hybrid FET circuit structure, b) circuit diagram of the two‐stage amplifier, c) transfer *I*–*V* curves at *V*_DS_ = 5 V from n‐type IZO FET (*W*/*L* = 4000/10 µm) and p‐type IDT–BT FETs (*W*/*L* = 4800/10 µm) integrated in the circuit (inset; an actual photograph of the fabricated amplifier), d,e) voltage transfer characteristics of an amplifier with untreated IZO FETs (d) and with UVNOI‐treated IZO FETs (e) measured by sweeping *V*_in_^+^ from 0 to 10 V with varied *V*_bias_ (4, 5, 6 V) and fixed positive drain voltage (VDD = 10 V), grounded source voltage (VSS = 0 V), and *V*_in_^−^ (5 V), f) differential voltage gain of the UVNOI‐treated amplifier extracted by ∂*V*_out_/∂*V*_in_.

To demonstrate the suitability of our performance and stability improved IZO FETs for integration into hybrid complementary circuits, we fabricated differential amplifiers combining n‐type, UVNOI‐treated IZO FETs with p‐type IDT–BT FETs on plastic foil, as depicted schematically in **Figure** **4a**. F4‐TCNQ was used as an additive to IDT–BT to improve its environmental stability. The detailed recipes and fabrication processes are described in the Experimental Section.

As illustrated in Figure 4b, the differential amplifier in our study consisted of a current mirror with common source configuration in the first and second stages, respectively. Two mirror transistors (T3 and T4) and the driving transistor (T7) in the second stage were made up of n‐type IZO, while p‐type IDT–BT was used for differential input switches (T1 and T2), current source transistor (T5) in the first stage, and a load transistor (T6) in the second stage. Figure 4d is the voltage transfer characteristics of the fabricated complementary circuit with untreated IZO, while Figure 4e shows the characteristics for UVNOI‐treated IZO.

For keeping a high voltage level in the output node (*V*_out_) at high *V*_in_ (>5 V) (Figure 4b), T7 should turn off without off‐leakage current. On the contrary, for maintaining low voltage in *V*_out_, T7 must turn on with high on‐current to ensure *V*_out_ connected to VSS without voltage drop. To achieve a high on‐current, the size of T7 should be large enough and a high voltage should be applied to the gate node of T7. The voltage is determined by current mirror output of the first stage. Two matching FETs (T3 and T4) in the current mirror are connected back‐to‐back such that they have the same *V*_GS_. T4 copies the current flowing through T3 when the same *V*_GS_ is applied to both T3 and T4. T3 is always in saturation mode and T4 may be in saturation, linear, or turn off mode. When T4 is in saturation mode, it can work properly as a current mirror. In this way, a high on‐current level must also be generated from T4 when it operates in saturation region that provides a low enough gate voltage to T7 allowing its complete off‐state. Therefore, high on‐current of the IZO FET is the most crucial determining factor for amplifier operation with a sharp switching characteristics and high gain. UVNOI‐treated IZO FETs exhibit sufficiently high on‐current (6 × 10^−4^ A at *V*_GS_ = 4 V) due to their improved FET mobility. As a result of the UVNOI treatment, *V*_out_ increases steeply from 0 V (*V*_out_low_) to 9.93 V (*V*_out_high_) at *V*_in_^+^ of 5 V with a high voltage gain of ≈100 V V^−1^, while the voltage transfer characteristics of the reference amplifier with untreated IZO exhibits a more gradual switching characteristics and a gain of just 53 V V^−1^. This may be due to the incomplete off‐state or insufficient current of T7 or T4.

**Figure 5:**
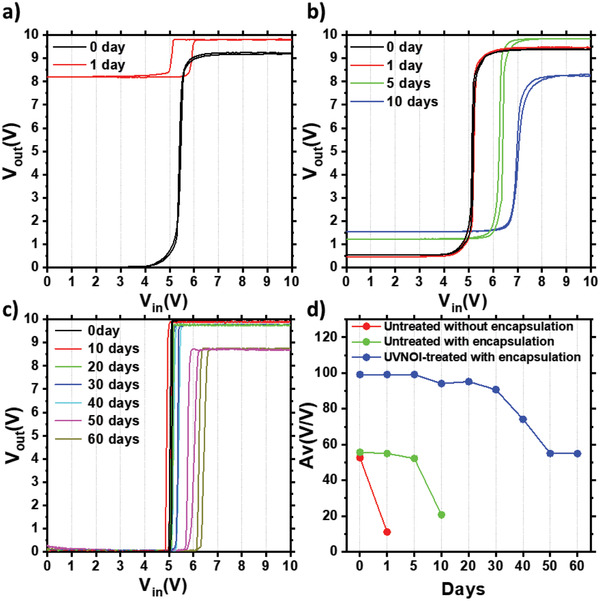
Air stability of hybrid differential amplifier. a) Untreated amplifier without encapsulation, b) untreated amplifier with encapsulation, c) UVNOI‐treated amplifier with encapsulation, and d) comparison of voltage gain reduction.

Finally, as an additional approach to further improve long‐term air stability of the circuits, we adopted an encapsulation approach on the top of the circuit using organic (CYTOP, UV‐curable polymer, and poly(ethylene terephthalate) (PET)) and inorganic (SiO_2_) multilayers, as described in the Experimental Section (Encapsulation). The inorganic layers provide a good barrier for blocking the diffusion of oxygen and water molecules.^[^ ^50^ ^]^ Without encapsulation, untreated devices exhibited degradation when stored in air for one day (**Figure** **5a**). The positive *V*_th_ shift or low on‐current of T4 causes a leakage current through T7 when it needs to be turned off, this results in a *V*_out_low_ increase. As displayed in Figure 5b, encapsulation impeded the diffusion of ambient contaminants and allowed the amplifier with untreated IZO devices to operate for about 10 days. Despite the extended lifetime with encapsulation, the transferring voltage (*V*_T_) was shifted from 5 to 7 V after 10 days and *V*_out_high_ and *V*_out_low_ were also degraded to 8.2 and 1.6 V, respectively. A more substantial improvement in the stability of the amplifier characteristics could be obtained by combining the UVNOI treatment on IZO FETs with the encapsulation on the top of whole circuit. As shown in Figure 5c, the lifetime of the UVNOI‐treated circuit (>60 days) is 6 times longer than that of the encapsulation‐only circuit. The *V*_T_ of the amplifier shifted by only 0.2 V after 40 days with stable *V*_out_high_ (9.87 V) and *V*_out_low_ (0.02 V). Even after 60 days, the circuit was still working though *V*_T_ shifted to 6.3 V. Figure 5d specifies the temporal variation of voltage gain extracted from the voltage transfer curves of the amplifiers. The lifetime in the treated circuit was almost double of the lifetime in the untreated circuit. We conclude that the UVNOI approach is not only effective in improving IZO FET stability and performance but also improves the lifetime and reliability of the hybrid complementary amplifier circuits.

## 3 Conclusions

We have demonstrated an effective UVNOI‐based surface treatment for significantly improving the FET stability and performance of solution‐processed IZO FETs. The UVNOI treatment reduces oxygen vacancy concentrations on the surface and the density of both shallow and deep trap states. The treatment allows an atomic level passivation of the surface, creating a more fully coordinated surface that is able to block ambient oxygen penetration into the bulk of the film. It prevents the trapping of electrons due to oxygen molecules adsorbed on the surface, leading to a large improvement in FET mobility and stability. This enables the fabrication of hybrid complementary amplifier circuits with higher gain and improved reliability and lifetime compared to untreated IZO devices. Our UVNOI treatment could be a simple, inexpensive and broadly applicable, effective treatment for enhancing the performance and stability of low‐temperature, solution‐processed oxide electronic devices.

## 4 Experimental Section

### Plastic Substrates

The FETs were fabricated on ultrathin, flexible polymer substrates. Polyimide (PI‐2555, HD Microsystems) was spin‐cast at 2000 rpm with 400 rpm dispersing step with a thickness of ≈3.7 µm on a rigid carrier, SiO_2_/p‐Si wafer. Before spin‐coating the resin, the carriers were cleaned with a standard cleaning process and treated with O_2_ plasma for the enhancement of the adhesion of polyimide (PI) on the carriers. The PI‐coated substrates were cured at 280 °C with a ramping step of 2.5 °C min^−1^ for 2 h 30 min in a N_2_ environment.

### Dual Buffer Layer

A 150 nm thick SiO_2_ was deposited using radio frequency (RF) sputter coating as a bottom buffer layer below the PI substrate coating. Before the buffer layer deposition, a thin delamination layer (<0.3 µm) of *N*‐methyl‐2‐pyrrolidone‐diluted PI‐2555 was coated at 5000 rpm and annealed at 280 °C for 3 h. It served to assist delamination of the flexible substrate from the carrier after all FET fabrication processes. After coating PI on the top of the bottom buffer layer, the same thick SiO_2_ was deposited as a top buffer layer. The top buffer layer protected PI substrates from solvent and water molecules during fabrication processes for FET layers above the substrates and prevented diffusion of degassed molecules residing in the substrate into the channel layers of FETs. The bottom buffer layer also enhanced the stability of FETs and PI substrate by blocking ambient molecules which could penetrate from the bottom surface of PI. Besides, the use of a symmetric substrate design with buffer layers on both sides of the PI substrate also ensured that the PI substrates remained flat without a curl after delamination.

### FET Circuits

For source and drain electrodes, Cr (2 nm)/Au (15 nm) was thermally evaporated on the buffer layer and patterned using photolithography. Following a gentle O_2_ plasma treatment (150 W, 65 sccm O_2_, 10 min) of the substrate, a hydrous IZO solution (42 mg of indium(III) nitrate hydrate (Sigma‐Aldrich, 99.999%) and 18 mg of zinc nitrate hexahydrate (Alfa Aesar, 99.998%) mixed in 1 mL of deionized water) was spin‐coated with 5000 rpm speed on the source‐/drain‐patterned substrates and annealed in two steps, at 150 °C for 30 s and at 275 °C for 2 h in ambient air condition. The coated IZO film was patterned using photolithography and wet etching in hydrochloric acid, then annealed again for 1 h at 180 °C in N_2_. In order to separate p‐type IDT–BT and n‐type IZO semiconductor region, the hydrophobic characteristics of CYTOP surface were utilized. 5 nm thick CYTOP was spin‐coated and annealed at 90 °C for 10 min followed by local etching with O_2_ plasma (250 W, 65 sccm O_2_, 40 s) through a suitable shadow mask to uncover confined, hydrophilic substrate regions for the IDT–BT p‐type channel. IDT–BT was dissolved in 1,2‐dichlorobenzene:chloroform (5:1) with a concentration of 10 g L^−1^. 10 wt% of F4‐TCNQ was mixed in IDT–BT solution before spinning at 3000 rpm. The IDT–BT was coated only on the etched regions by selective dewetting from the CYTOP‐covered regions. The films were then annealed at 90 °C for 10 min in N_2_. 25 nm thick CYTOP and 210 nm thick P(VDF–TrFE–CFE) were applied for the dual layer of gate dielectric to enable low‐voltage driving devices. The low‐*k* dielectric, CYTOP was spin‐coated at 5000 rpm and annealed at 90 °C. Then, 1 nm thick AlO*_X_* was deposited to improve the wetting characteristics of the CYTOP layer and P(VDF–TrFE–CFE) in *n*‐butyl acetate (40 g L^−1^) was spin‐coated at 1500 rpm and annealed at 60 °C for 2 h. 35 nm thick Al was thermally evaporated on the top of the dielectric for a top‐gate electrode.

### Environmental Conditions for Air Storage

Air‐stability evaluation was conducted by exposing IZO FETs to ambient air (21% of oxygen) with 35–40% relative humidity (RH) at room temperature without encapsulation layers.

### Various Literature Treatments for Stability

For lithium doping in IZO film, 16.8 mg of lithium nitrate (Sigma‐Aldrich, 99.99%) was added in the mixture of indium(III) nitrate hydrate and zinc nitrate hexahydrate in 1 mL of deionized water. For F‐doped IZO, 34.4 mg of indium(III) fluoride trihydrate (Sigma‐Aldrich, 99%) was mixed with 14.5 mg of zinc nitrate hexahydrate in deionized water. The solutions were mixed overnight at room temperature and coated on substrates using the same recipe for undoped IZO film described above. For hydrogen peroxide treatment, 30% H_2_O_2_ was coated on deposited IZO film and annealed at 120 °C for 5 min. UV–ozone treatment was carried out on IZO film with 365 nm UV optical source (365 nm, 22 mW m^−2^) for 15 min and ozone generating system (PSDP‐UVT, Novascan Co.) for 30 min. UV–O_2_ plasma treatment was also performed with weak O_2_ plasma (25 W, 65 sccm O_2_) for 10 min after UV light illumination on IZO film for 5 min.

### UVNOI Treatment

After forming and patterning IZO film on PI substrate, the films were subjected to 20 min UV illumination (365 nm) immediately followed by NOI treatment with a high density negative ion generator (≈8.5 million ions cc^−1^ with no ozone; ozone density < 0.001 ppm, Ion trading‐Universal Plan Co. Ltd.) in a chamber with 1 LPM O_2_ flow for various time periods (20–40 min). After the treatment, dielectric layer was deposited without delay and other FET fabrication processes on the top of IZO were completed.

### Encapsulation

Multilayer encapsulation of CYTOP (50 nm)/SiO_2_ (100 nm)/CYTOP (50 nm) was formed by spin‐coating and RF sputtering on the top of gate metal immediately followed by applying UV‐curable polymer, NOA74 (Norland Co.) layer (≈5 µm) covered by PET (70 µm). Then, it was cured with 365 nm UV light illumination for 10 min.

### Delamination

After the FET fabrication and encapsulation processes, the flexible substrates were delaminated from rigid SiO_2_ carriers. The four side edges of PI substrates were cut and FET region in the middle was mechanically delaminated before testing circuits.

### Electrical Characterization

All FET characteristics and contact resistance were measured at room temperature in air condition using HP4155C Semiconductor Parameter Analyzer and the characterizations of differential amplifiers were performed with Agilent B1500A in N_2_ glove box. For the analysis of air stability, the amplifiers were stored in air only except for measuring the characteristics. The FE mobility values were extracted from drain current characteristics of FETs as the saturation mobility, $\mu_{\mathrm{sat}}\;=\;2{\left(\frac{\partial {\left(I_{\mathrm{DS}}\right)}^{\frac{1}{2}}}{\partial V_{\mathrm{GS}}}\right)}^{2}/\left(\frac{C_{\mathrm{ch}}W}{L}\right)$, when *C*_ch_, *W*, and *L* are the capacitance, width (1000 µm), and length (20 µm) of FET channel, respectively. Effective mobility values (*μ*_eff_  =  *r*  ×  *μ*_sat_) were extracted by multiplying the values with reliability factor, $r={\left(\frac{{\left({I_{\mathrm{DS}}}^{\max}\right)}^{1/2}-\;{\left({I_{\mathrm{DS}}}^{0}\right)}^{1/2}}{{V_{\mathrm{GS}}}^{\max}}\right)}^{2}/{\left(\frac{\partial {\left(I_{\mathrm{DS}}\right)}^{1/2}}{\partial V_{\mathrm{GS}}}\right)}^{2}\;$   where *I*_DS_^max^ is the maximum source–drain current reached at the maximum gate voltage (*V*_GS_^max^) and *I*_DS_^0^ denotes the current at *V*_GS_ = 0.^[^ ^39^ ^]^

### Optical Characterization

The PDS samples were spin‐coated on a 11 mm quartz spectrosil and annealed at 150 °C for 30 s and at 275 °C for 2 h in air. The absorbance was measured in a spectrophotometer from UV to visible wavelengths.

## Conflict of Interest

The authors declare no conflict of interest.

## Supporting information

Supporting Information

## Data Availability

The data that support the findings of this study are openly available at https://www.data.cam.ac.uk/repository.
