# Supplementary material for: Surface Passivation Treatment to Improve Performance and Stability of Solution‐Processed Metal Oxide Transistors for Hybrid Complementary Circuits on Polymer Substrates
Source: Adv Sci (Weinh). 2021 Oct 20;8(23):2101502. doi: 10.1002/advs.202101502 (PMC8655209; doi:10.1002/advs.202101502)
Supplement: Supplementary file 1 — Supporting Information [file ADVS-8-2101502-s001.pdf]

© 2021 Wiley-VCH GmbH

ADVANCED SCIENCE Open Access

Supporting Information

for *Adv. Sci.*, DOI: 10.1002/advs.202101502

Surface Passivation Treatment to Improve Performance and Stability of Solution-Processed Metal Oxide Transistors for Hybrid Complementary Circuits on Polymer Substrates

Moon Hyo Kang, John Armitage, Zahra Andaji-Garmaroudi, and Henning Sirringhaus**

Supporting Information

Surface Passivation Treatment to Improve Performance and Stability of Solution-Processed Metal Oxide Transistors for Hybrid Complementary Circuits on Polymer Substrates

Moon Hyo Kang, John Armitage, Zahra Andaji-Garmaroudi, and Henning Sirringhaus**

Optoelectronics Group, Cavendish Laboratory, University of Cambridge, J J Thomson Avenue, Cambridge CB3 0HE, UK
E-mail: mhk29@cam.ac.uk, hs220@cam.ac.uk

The stability of metal oxide thin film and FETs is related to oxygen vacancies. Several approaches have been used to control oxygen vacancies or preclude ambient molecules to diffuse inside the film as enumerated in **Table S1**.

Table S1. Approaches for the stability of metal oxide FETs.

Materials	Film formation	Treatments for stability	μ_{FE} ($cm^2/V{\cdot}s$) before treatment	μ_{FE} ($cm^2/V{\cdot}s$) after treatment	Stability after treatment - ΔV_{th} (V)	Ref.
ZnO	Solution process	Film thickness control	1.5	11	+4 (30days in air ambient)	[1]
ZnO	ALD	TiO_2 passivation	16.6	20.2	+1 (1200hours in air ambient)	[2]
InGaZnO	Sputtering	Nitrogen-doped bilayer	6.7	7.3	-2.1 (7days stored in 50% RH*1)	[3]
InGaZnO	Sputtering	Post annealing	N/A	4.7	-4.4 (9days store in 50% RH)	[4]
InZnO	Solution process	High pressure annealing	0.15	4.85	~+5 (1000s PBS*2) ~-0.5 (1000s NBS*3)	[5]
InGaZnO	Sputtering	H_2O_2 doping	9.1	17.5	-4.6 (3600s NBIS*4)	[6]
InZnO	Solution process	Li doping	0.13	2.3	+4.5 (1000s PBS)	[7]
InZnO	Solution process	Li doping	0.06	12	~+0.9 (1000s PBS)	[8]
InZnO	Solution process	F doping	N/A	4.1	+0.45 (3600s PBS)	[9]

InGaZnO	Sputtering	Oxygen-pressure control	10.8	9.2	+5.6 (10000s PBS)	[10]
InGaZnO	Sputtering	Tantalum catalytic layer	18.1	54	+2.56 (3600s PBS)	[11]
InGaZnO	Sputtering	Long post-annealing	14.7	17.9	~-1.7 (1000s NBIS)	[12]
InZnO	Solution process	Acid catalytic reaction	13.0	8.1	+0.7 (3600s PBS) -0.6 (3600s NBS)	[13]
InZnO	Solution process	Photocatalytic reaction treatment	2.8	7.8	+2.91 (1000s PBS), -2.75 (1000s NBIS)	[14]
InGaZnO	Sputtering	Additional oxygen flow	7.16	12.0	+2.95 (10000s PBS), -8.99 (10000 NBIS)	[15]
InZnTO	Sputtering	Controlled crystallization	22.4	39.2	+1.52 (3600s PBS), -0.13 (3600s NBS)	[16]

**1RH: Relative Humidity, *2PBS: Positive Bias Stress, *2 NBS: Negative Bias Stress, *3 NBIS: Negative Bias illumination Stress*

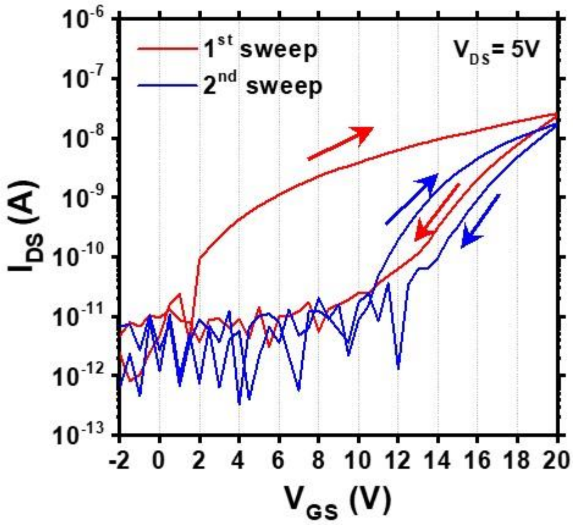

Figure S1. Transfer characteristics of an untreated IZO FET after stored in air for 10 days. The gate bias was swept from -2 V to 20 V and repeated.

As demonstrated in **Figure S1**, V_{th} of the untreated IZO FET was shifted during the 1st gate bias sweep and it could not be recovered at the 2nd sweep. During the 1st sweep, some of the accumulated electrons were trapped by oxygen adsorbing onto the surface of the films from ambient air leading to hysteresis. When V_g swept back

from 20 V to -2 V, current level was kept lower than the level at the previous forward sweep from -2 V to 20 V. At the 2nd sweep, the low current level essentially remained. This suggests that the positive gate bias leads to deep trapping of electrons in the presence of oxygen.

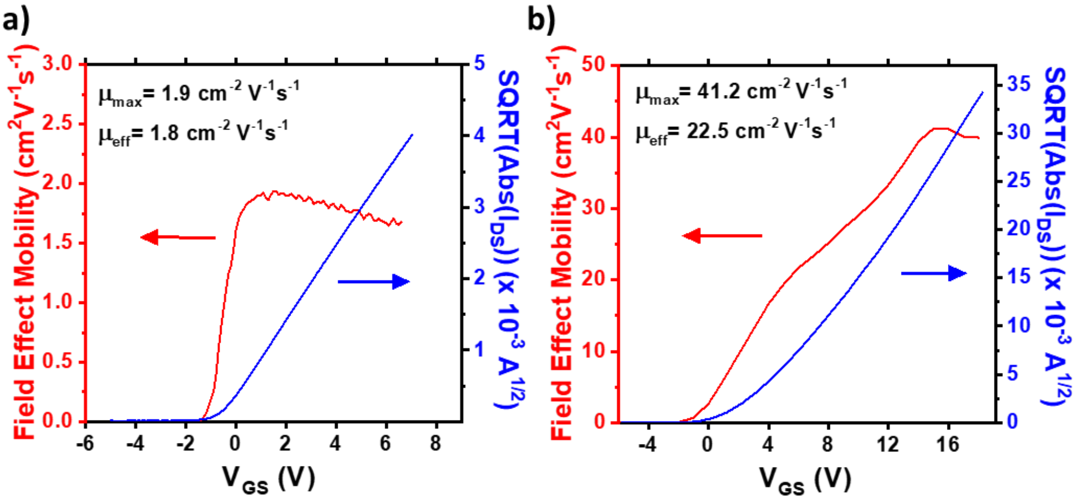

Figure S2. Field effect mobilities of a) untreated, and b) UVNOI-treated IZO FETs which were extracted from square root drain current.

Figure S2 compares the field effect (FE) mobility of UVNOI treated IZO FET with an untreated IZO FET. The FE mobility was extracted from the square-root of the saturated drain current using the equation described in experimental section in the manuscript. The mobility of untreated FETs that we fabricated using solution-processed IZO film showed 1.9 cm^{2}V^{-1}s^{-1} as the maximum value at 1.8 V (**Figure S2 a)**). With UVNOI treatment, the mobility curve showed an increasing trend with gate voltage beyond 7 V and thus, the measurement for UVNOI treated FET was repeated with higher voltage as far as 18 V (**Figure S2 b)**) in order to find the maximum mobility, 41.2 cm^{2}V^{-1}s^{-1} at 15 V.

However, in order to obtain a more reliable measurement of mobility we also extracted effective mobility values using the reliability factor,

$r = \left(\frac{(I_{DS}{}^{max})^{\frac{1}{2}} - (I_{DS}{}^{0})^{\frac{1}{2}}}{V_{GS}{}^{max}} \right)^2 \Bigg/ \left(\frac{\partial (I_{DS})^{\frac{1}{2}}}{\partial V_{GS}} \right)^2$, when $I_{DS}{}^{max}$ is the maximum source-drain current reached at the maximum gate voltage ($V_{GS}{}^{max}$) and $I_{DS}{}^{0}$ denotes the current at V_{GS} = 0.[17] The effective mobility of untreated and UVNOI-treated IZO were 1.8 $cm^2V^{-1}s^{-1}$ and 22.5 $cm^2V^{-1}s^{-1}$, respectively.

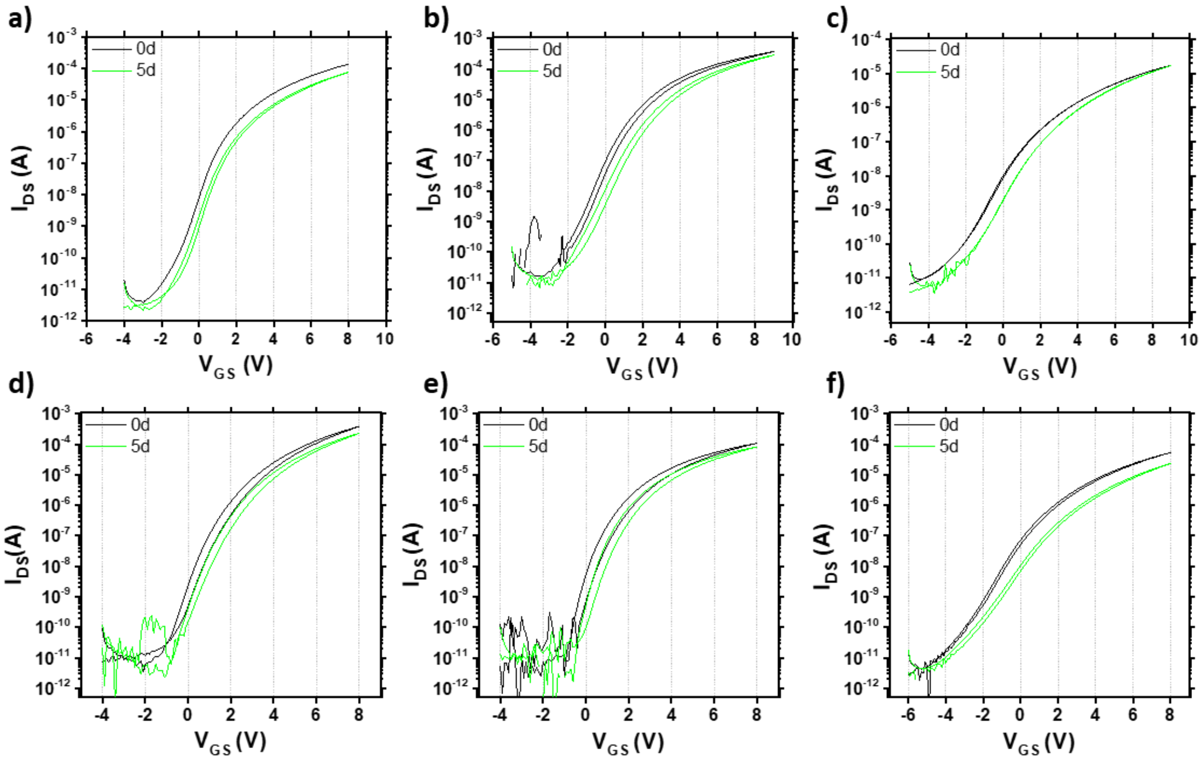

Figure S3. Air stability of UVNOI-treated IZO FETs. All samples (a)-f)) were fabricated with the same condition.

In order to confirm the reproducibility of device fabrication and stability, 6 batches of FETswere fabricated with the same treatment condition (20 min. UV illumination and 40 min. NOI treatment) on different days The fabricated FETs were stored in air ambient condition (21% of O_2, 35-40% RH) for 5 days and the I_D-V_G characteristics were measures before and after storing the samples. There was some variation in performance from batch to batch as would be expected for a research environments, but the V_{th} shift of the treated samples was consistently and averaged to 0.54 ± 0.12 V after 5 days.

Table S2. Comparison of electrical performances of FETs before and after stored in ambient air condition.

Treatment	μ_{FE} *1 (cm^2/V·s) Before	μ_{FE} *1 (cm^2/V·s) After	V_{th} (V) Before	V_{th} (V) After	I_{on}/I_{off} Before	I_{on}/I_{off} After
Untreated	1.8	0.007 After 2 days	-0.4	4.6 After 2 days	10^5	10^2 After 2 days
Li-doped	0.008	0.0036 After 2 days	0.5	3.9 After 2 days	10^3	10^3 After 2 days
F-doped	1.2	0.06 After 5 days	0.6	2.5 After 5 days	10^7	10^5 After 5 days
H_2O_2 treated	0.07	0.01 After 2 days	1.7	3.1 After 2 days	10^5	10^4 After 2 days
UV+ozone treated	7.1	0.16 After 4 days	1.6	3.8 After 4 days	10^8	10^6 After 4 days
UV+plasma treated	5.3	0.06 After 5 days	-0.8	2.3 After 5 days	10^6	10^5 After 5 days
UVNOI treated	22.5	12.0 After 5 days	1.0	1.5 After 5 days	10^8	10^8 After 5 days

*1 *μ_{FE}: Feield-effect mobility caculated with reliability factor.*

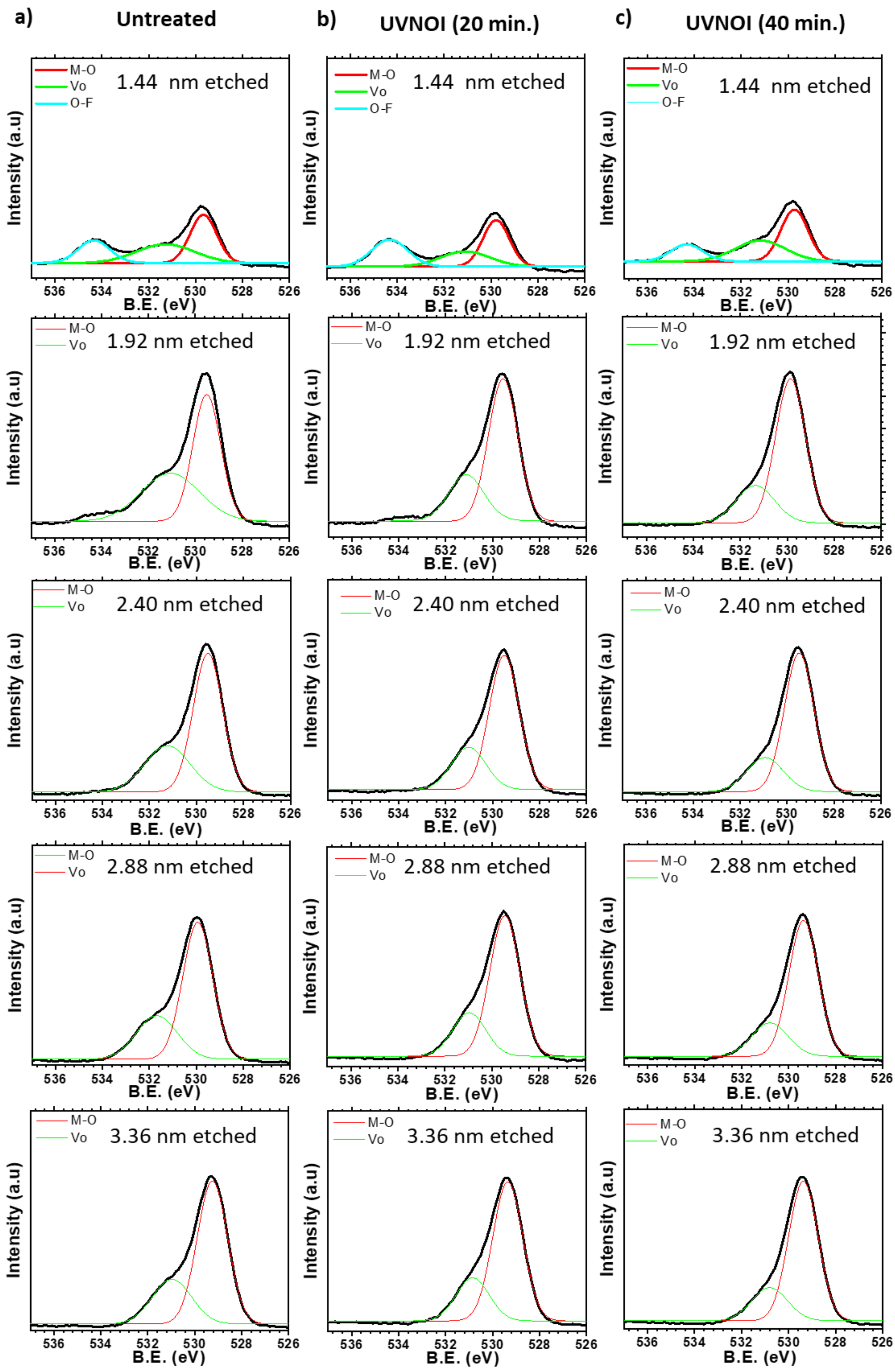
a)
Untreated
b)
UVNOI (20 min.)
c)
UVNOI (40 min.)
M-O
Vo
O-F
1.44 nm etched
1.92 nm etched
2.40 nm etched
2.88 nm etched
3.36 nm etched
Intensity (a.u)
B.E. (eV)
536
534
532
530
528
526

Figure S4. XPS 1Os peaks from a) Untreated, b) UVNOI treated (20 minutes), and c) UVNOI treated (40 minutes). Five graphs of each column are from each sample (a), b), and c)) whilst each row shows different ion-etching depth.

XPS O1s analysis was performed through a variation of film depth with Ar-ion etching. The O1s peak was acquired from untreated and UVNOI treated IZO films (two different NOI treatment times: 20 and 40 minutes) as demonstrated in **Figure S4**. The graphs in the same column show the same sample and the rows of the graphs are from the different samples (a) untreated, b) 20 min UVNOI-treated, and c) 40 min UVNOI-treated IZO) measured at the same etching depth (1.44 – 3.36 nm).

Due to the X-ray penetration depth (> 1nm), the IZO film was covered with CYTOP (4-5 nm) to measure the depth profile of O1s from genuine surface of IZO. Then, the film was etched and the measurements were repeated from the top of CYTOP to the bulk of IZO. The Voigt-convolution fitted O1s peaks have three oxygen bonding components (M-O, V_O, and O-F) as shown in **Figure S4.** Through the CYTOP layer thickness, no M-O bonding peak (~529.5 eV) [8] was observed and it appeared at the etching depth of 1.44 nm together with a small O-F peak (~535 eV)[18]. This indicates that the XPS measurement on 1.44 nm was achieved from the interface between CYTOP and IZO. At the depth of 1.92 nm, the peak with fluorine disappeared and high M-O bonding peak was observed exhibiting the top surface of IZO. The oxygen vacancy peak was observed as a hump on the left side of the peak at ~531 eV which is consistent with previous findings.[19,20] On the surface (1.92 nm depth), a large vacancy hump was observed from the untreated IZO film, which gradually reduced when measuring deeper in the bulk. This variation demonstrates that in untreated films the IZO surface has more oxygen vacancies than the bulk.[21,22]

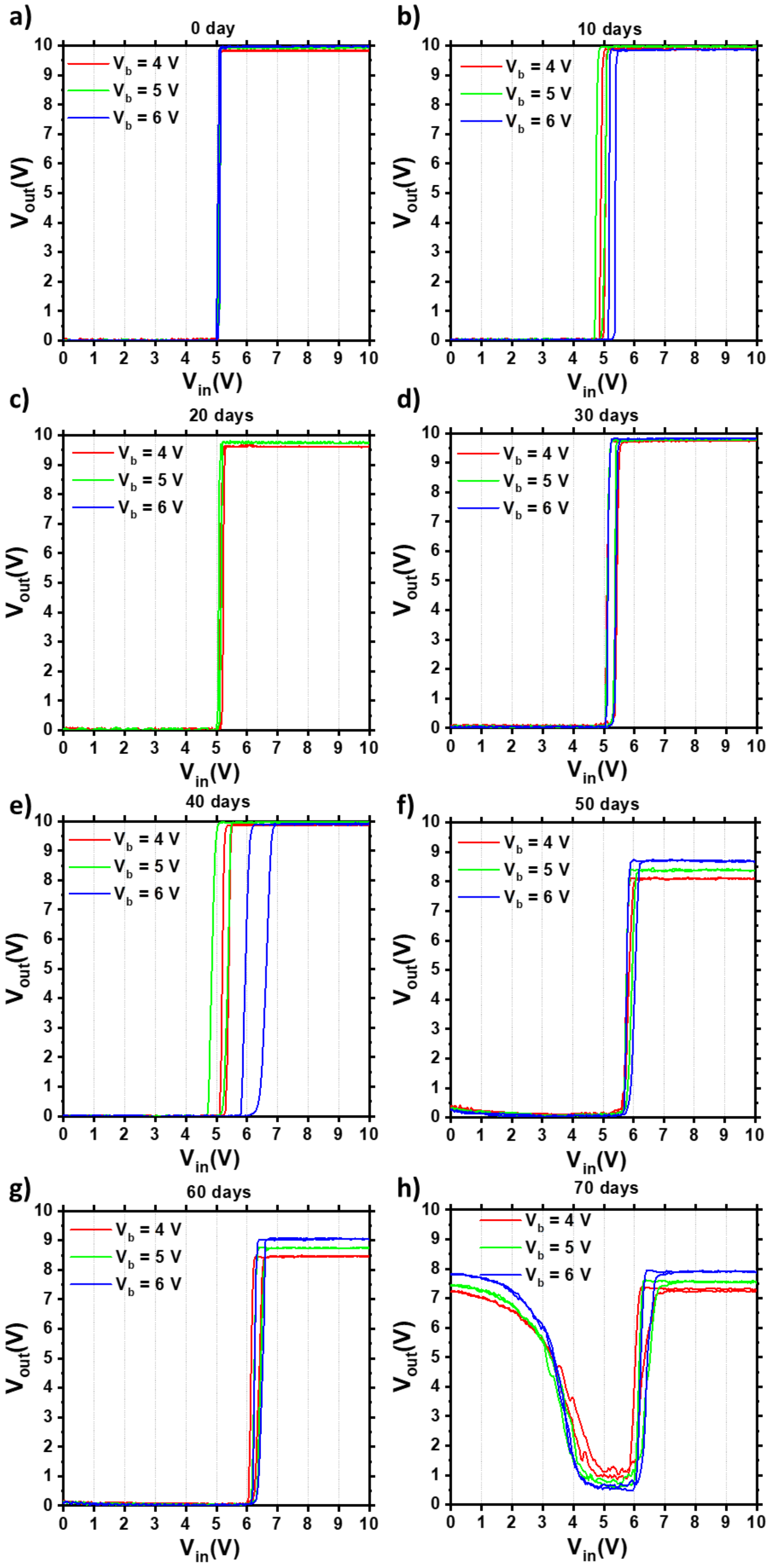

Figure S5. Air-stability of hybrid complementary amplifier with UVNOI treatment. The voltage transfer characteristics of an amplifier was measured after a) 0 day, b) 10 days, c) 20 days,

d) 30 days, e) 40 days, f) 50 days, g) 60 days, and h) 70 days while storing the circuit in air ambient condition.

References

[1] M. Ortel, S. Pittner, V. Wagner, Journal of Applied Physics **2013**, 113, 154502.
[2] Y.-Y. Lin, C.-C. Hsu, M.-H. Tseng, J.-J. Shyue, F.-Y. Tsai, ACS Applied Materials & Interfaces **2015**, 7, 22610.
[3] P. Liu, Y. Chou, L. Teng, F. Li, C. Fuh, H. D. Shieh, IEEE Elec. Dev. Lett. **2011**, 32, 1397.
[4] C.-S. Fuh, P.-T. Liu, Y.-T. Chou, L.-F. Teng, S. M. Sze, ECS Journal of Solid State Science and Technology **2012**, 2, Q1.
[5] Y. S. Rim, W. H. Jeong, D. L. Kim, H. S. Lim, K. M. Kim, H. J. Kim, Journal of Materials Chemistry **2012**, 22, 12491.
[6] H. J. Kim, Y. J. Tak, S. P. Park, J. W. Na, Y.-g. Kim, S. Hong, P. H. Kim, G. T. Kim, B. K. Kim, H. J. Kim, Scientific Reports **2017**, 7, 12469.
[7] J. H. Park, J. Y. Oh, H. K. Baik, T. I. Lee, Journal of Materials Chemistry C **2015**, 3, 6276.
[8] S.-Y. Han, M.-C. Nguyen, A. H. T. Nguyen, J.-W. Choi, J.-Y. Kim, R. Choi, Thin Solid Films **2017**, 641, 19.
[9] J.-S. Seo, J.-H. Jeon, Y. H. Hwang, H. Park, M. Ryu, S.-H. K. Park, B.-S. Bae, Scientific Reports **2013**, 3, 2085.
[10] J. H. Park, Y.-g. Kim, S. Yoon, S. Hong, H. J. Kim, ACS Applied Materials & Interfaces **2014**, 6, 21363.
[11] Y. Shin, S. T. Kim, K. Kim, M. Y. Kim, S. Oh, J. K. Jeong, Scientific Reports **2017**, 7, 10885.
[12] M. D. H. Chowdhury, J. G. Um, J. Jang, Applied Physics Letters **2014**, 105, 233504.
[13] H. Park, Y. Nam, J. Jin, B.-S. Bae, Journal of Materials Chemistry C **2014**, 2, 5998.
[14] J. K. Kang, S. P. Park, J. W. Na, J. H. Lee, D. Kim, H. J. Kim, ACS Applied Materials & Interfaces **2018**, 10, 18837.
[15] C. S. Yoon, H. T. Kim, M. S. Kim, H. Yoo, J. W. Park, D. H. Choi, D. Kim, H. J. Kim, ACS Appl Mater Interfaces **2021**, 13, 4110.
[16] N. On, B. K. Kim, Y. Kim, E. H. Kim, J. H. Lim, H. Hosono, J. Kim, H. Yang, J. K. Jeong, Scientific Reports **2020**, 10, 18868.
[17] H. H. Choi, K. Cho, C. D. Frisbie, H. Sirringhaus, V. Podzorov, Nature Materials **2018**, 17, 2.
[18] T. F. S. Inc., in *Elements table*, thermofisher.com, **2013-2020**.
[19] C. Chen, G. Chen, H. Yang, G. Zhang, D. Hu, H. Chen, T. Guo, Journal of Materials Chemistry C **2017**, 5, 9273.
[20] J. Socratous, K. K. Banger, Y. Vaynzof, A. Sadhanala, A. D. Brown, A. Sepe, U. Steiner, H. Sirringhaus, Adv. Funct. Mater. **2015**, 25, 1873.

[21] J. Carrasco, N. Lopez, F. Illas, H.-J. Freund, The Journal of chemical physics **2006**, 125, 074711.
[22] E. V. Stefanovich, T. N. Truong, The Journal of chemical physics **1995**, 102, 5071.
